# Trends in Microalgae Incorporation Into Innovative Food Products With Potential Health Benefits

**DOI:** 10.3389/fnut.2018.00058

**Published:** 2018-07-31

**Authors:** Martín P. Caporgno, Alexander Mathys

**Affiliations:** Laboratory of Sustainable Food Processing, Institute of Food Nutrition and Health IFNH, ETH Zurich, Zurich, Switzerland

**Keywords:** bioactive compounds, innovative food products, microalgae, peptides, proteins

## Abstract

Microalgae have demonstrated potential to meet the population's need for a more sustainable food supply, specifically with respect to protein demand. These promising protein sources present several advantages over other currently used raw materials from an environmental point of view. Additionally, one of the main characteristics of microalgae is the production of bioactive compounds with potential benefits for human health. Microalgae exploitation as a source of protein (bulk protein) and other valuable products within the food industry still presents some drawbacks, mainly because of the underdeveloped technologies and processes currently available for microalgae processing. The systematic improvement of the technology readiness level (TRL) could help change the current situation if applied to microalgae cultivation and processing. High maturity in microalgae cultivation and processing technologies also requires improvement of the economy of scale and investment of resources in new facilities and research. Antioxidative, antihypertensive, immunomodulatory, anticancerogenic, hepato-protective, and anticoagulant activities have been attributed to some microalgae-derived compounds such as peptides. Nevertheless, research on this topic is scarce and the evidence on potential health benefits is not strong. In the last years, the possibility of using microalgae-derived compounds for innovative functional food products has become of great interest, but the literature available mainly focuses more on the addition of the whole cells or some compound already available on the market. This review describes the status of utilising microalgae as an ingredient in innovative food products with potential health benefits.

## Food supply and human nutrition

The amount of food currently produced must double to meet the needs of the expected population of around 9.8 billion people by 2050 (1, 2). The existence of a significantly increasing protein demand was reported years ago (3). Nowadays, approximately one billion people have inadequate protein intake; furthermore, conventional sources of protein are predicted to be insufficient (4). Plant-based proteins account for the majority of protein intake worldwide used for food and feed. In the EU, animal-based proteins are consumed in greater quantity than plant-based proteins^1^; however, concerns about health and environmental issues as well as animal welfare could give a boost to plant-based sources.

Food production accounts for between 20 and 30% of the total environmental impact (5) and for almost 30% of global greenhouse gas emissions (6). More than 80% of the protein imported in Europe for livestock nutrition comes from non-European countries, much of it from non-sustainable and environmentally damaging sources (7). New food technologies and products may help to reduce the environmental impact of people's eating behaviour (8). Changing dietary patterns could also significantly improve this situation (9) and simultaneously reduce the environmental impact. Taking Germany as an example, switching from an omnivore to an ovo-lacto-vegetarian diet would reduce food-based greenhouse gas emissions by one third, and by half when changing to a vegan diet (10). In the US, a change to an ovo-lacto-vegetarian diet would reduce energy consumption, even if both meat-based and plant-based diets are challenging in terms of energy, land, and water consumption (11). Rosi et al. (12) suggest that in addition to the type of diet, environmental impact is related to individual dietary habits, i.e., intake of various types of food and the frequency of intake in terms of times per day or week. Additionally, indicators of sustainability such as nutrition, environment, food affordability and availability, sociocultural well-being, resilience, food safety, and waste, considerably differ between high-income and low-income countries (9, 13).

## Microalgae as a source of proteins and other nutritional components

As mentioned above, plant-based proteins are currently the main source of protein for food and feed. Expanding the cultivation area, changing the cropping frequency, and boosting yields could help meet the increasing food demand; however, crop production may be approaching a ceiling in terms of optimisation. Additionally, these practices could seriously deepen existing environmental problems derived from current cultivation systems, i.e., land degradation, loss of biodiversity, and deforestation (14). Animal-based proteins depend on the supply of appropriate and cost-effective plant-based proteins for feeds (15). Microalgae have arisen as a promising sustainable alternative protein source. By the middle of this century, algae may account for 18% of protein sources in a more diverse market^2^. However, aspects related to food safety of algae are not well-known, namely the presence of contaminants, allergens, or hazardous substances generated during microalgae processing. Hence, the estimated time to market of microalgae and other protein sources differs (16).

*Nostoc, Arthrospira* (usually denoted as *Spirulina* in the market), and *Aphanizomenon* are protein-rich microalgae that have been part of the human diet since thousands of years ago (17). Spanish chroniclers observed Aztecs consuming a blue-green cake made from *Arthrospira* (18). Exploiting microalgae for food and biochemical applications was suggested in 1952 at the Algae Mass-Culture Symposium, even if some progress had been made in the early 1940s. The first facilities for commercial production of *Chlorella* were developed in Japan, whereas Mexico pioneered *Arthrospira* cultivation in the 1970s (17). Although the number of microalgae species in nature is estimated between 200,000 and 800,000, only a few are used in food applications (19). In the USA, the regulatory status of algae products and additives is under the responsibility of the Food and Drug Administration (FDA), which can assign GRAS status (Generally Recognized as Safe) to a product^3^. In Europe, the competent authority of a member state makes a first assessment of a new product, which is later authorised by the European Commission (EC) if no objections are made by member states. In case of objections, the European Food Safety Authority (EFSA) is responsible for carrying out the safety assessment of the novel foods (16).

Microalgae as a source of bulk proteins is quite a new idea. Microalgae-based proteins could significantly contribute to meet the population's need for protein, with several advantages over other currently used protein sources. Microalgae-based proteins have low land requirements compared to animal-based proteins: <2.5 m^2^ per kg of protein (20) compared to 47–64 m^2^ for pork, 42–52 m^2^ for chicken, and 144–258 m^2^ for beef production (21). Land requirements are also lower than for some other plant-based proteins used for food and feed such as soybean meal, pea protein meal, and others (22). Furthermore, the usage of non-arable land for cultivation, minimal fresh water consumption, the possibility of growing in seawater, and the potential replacement of non-sustainable soy imports are some advantages of algae over other plant-based protein sources (23). When it comes to quality, *Chlorella* and *Arthrospira* accumulate high-quality proteins, having both species well-balanced amino acid profiles according to the WHO/FAO/UNU recommendations regarding human's requirements of essential amino acids (EAAs) (24, 25). The amino acid profiles of both species are similar to other conventional protein sources such as eggs and soybean (24). In general, microalgae as plants are deficient in sulphur-containing amino acids methionine and cysteine (24); however, some microalgae supplements showed to be deficient in other amino acids (26). A comparison between the amino acids profiles of several algal products, including commercially available products such as *Chlorella* pills and *Arthrospira* flakes, showed that some supplements can provide high amounts of EAAs. It is worth mentioning that the cultivation conditions or sources of the biomass used for these products can lead to differences in the amino acids profiles of the products (26). Nevertheless, during consumption, protein bioavailability becomes important. At this point, three different concepts need to be explained: bioaccessibility, bioavailability, and bioactivity (27). The bioaccessibility, usually evaluated by *in vitro* tests, represents the fraction of the compound released from the food matrix becoming available for absorption. Afterwards, the compounds may reach the systemic circulation and being utilised, which is referred to as bioavailability. The bioavailability is determined by *in vivo* tests. Finally, the bioactivity of a compound describes the physiological response, e.g., antioxidative, antihypertensive or anticancerogenic activities. The bioactivity can be evaluated *in vivo, ex vivo*, and *in vitro*. Based on these definitions, a compound can be considered bioaccessible, but not necessarily bioactive. Protein bioavailability from whole microalgae cells could be enhanced by applying pre-treatments to disrupt cell walls, which hinder degradation (25).

Besides proteins, microalgae are source of several valuable compounds with health benefits such as carbohydrates, polyunsaturated fatty acids, essential minerals, and vitamins (24, 25, 28), which can increase the nutritional value of food products upon incorporating. Polysaccharides and oligosaccharides are promising compounds with potential health benefits, arising attention in terms of prebiotic applications (29–31). This association is based on the first definition of prebiotics as “non-digestible food ingredient that beneficially affects the host by selectively stimulating the growth and/or activity of one or a limited number of bacteria in the colon, and thus improves host health,” given by Gibson and Roberfroid (30). *Arthrospira, Chlorella* and *Nannochloropsis* are not only a good source of proteins, but have been reported as important sources of polysaccharides or oligosaccharides, being proposed as potential prebiotic candidates (28–30). Lipids, in particular long-chain polyunsaturated omega-3 fatty acids (ω-3 PUFAs), have been also suggested as valuable compounds with health benefits that can be incorporated into food products. α-linolenic acid (ALA; 18:3 n-3), eicosapentaenoic acid (EPA; 20:5 n-3), docosapentaenoic acid (22:5 n-3) and docosahexaenoic acid (DHA; 22:6 n-3) are some of the most important ω-3 PUFAs with health benefits for humans (28). EPA and DHA were for instance associated with the prevention or amelioration of cardiovascular or renal diseases. These long-chain EPA and DHA, considered as essential dietary nutrients, can be produced only by plants, thus consumers must incorporate them into their diet (32). EPA and DHA can be also synthesised from ALA, but the process is very inefficient in humans and the fish oil is still the main source of EPA and DHA commercially available (32). Microalgae are a valuable source of ω-3 PUFAs. *Arthrospira, Chlorella, Dunaliella, Haematococcus, Schizochytrium, Porphyridium cruentum*, and *Crypthecodinium cohnii* have GRAS status (33). Most of the commercially available biomass is marketed as pills and capsules. *Arthrospira* and *Chlorella* are commonly consumed as food supplements, *Tetraselmis chuii* as a seafood flavouring agent, and the diatom *Odontella aurita* is consumed as a food supplement as it is rich in EPA (16). Some microalgae-derived products marketed are: β-carotene from *Dunaliella*, DHA from *C. cohnii*, and the blue colorant phycocyanin from *Arthrospira* (33, 34).

## Marketability of microalgae-based products

Large-scale commercialisation of microalgae-based products does however present some drawbacks. Algae-based bulk products currently on the market are mainly derived from seaweed or algae harvested from natural habitats; existing large-scale facilities allocate their products to aquaculture or producing high-value compounds (22). There are also barriers related to getting new products approved by regulatory authorities (16). In addition, a proven market demand and market value for microalgae-based products is difficult to ensure, especially for food products. It is true that investors seek opportunities with long-term market demand before deciding on investments, which combined with research on microalgae cultivation, are essential to develop more sustainable processes for competitive markets. For example, if microalgae production is compared to other crop-based protein sources, the latter have been grown for years, therefore cultivation and processing are optimised. Ruiz et al. (35) estimated that by increasing the size of a production facility from 1 to 100 hectares, cultivation (autotrophic) and biorefining costs per kg of biomass could be reduced ten times. However, changes in microalgae production processes are not easy to implement. The composition of the biomass, which can define its incorporation into food products, depends on the microalgae species but also on cultivation parameters. Different impacts of the cultivation systems currently existing and the operating parameters on the biomass productivity have been reviewed in detail (36, 37). Harvesting and dewatering steps strongly affect the biomass production costs; the characteristics of the harvesting and dewatering process itself, the cultivation systems and the size of the facility need to be considered (38). The systematic improvement of the technology readiness level (TRL) could help to achieve higher maturity in the development of technology for microalgae cultivation and processing (39), which might result in an improved economy of scale.

Biorefining costs represents between 20 and 40% of the total production costs when processing common biomass, but they increase up to 50–60% for microalgae due to underdeveloped technologies and processes currently available (40). Cell disruption, extraction, and fractionation, among other processes, could significantly reduce costs if optimised. Much research has been done aiming to reduce the biomass costs for biofuels production, which should be below 1 € per kg for competitive biofuels (41), and the results have significantly contributed to optimise the technologies and processes currently available for microalgae cultivation and processing. Besides costs, aspects related to the sustainability of the whole value chain for microalgae-based proteins could be improved; when processed for meat substitutes, a high-moisture extruded *Chlorella* (grown heterotrophically) resulted in a more environmentally sustainable product than pork and beef (22), with current TRL and economy of scale for microalgae production.

Products such as β-carotene, astaxanthin, and phycocyanin cost between hundreds to thousands of euros per kg depending on their purity (36, 42), and the high premium they attract on the market make them very appealing to businesses. On the other hand, the whole microalgae cells as food supplements are on the market below 40 € per kg (23, 34, 36). In this scenario, microalgae-based products with biofunctional compounds and higher selling prices than the common food supplements could improve their economic feasibility; the higher selling prices would allow covering higher costs derived from new cultivation and processing technologies. Microalgae production either as a final product or as biomass for microalgae-based products is an expanding sector, thus large enterprises and start-ups show interest worldwide. Sharma and Sharma (43) listed some of the most important companies currently involved in the market of microalgae and microalgae-based products.

**Figure 1:**
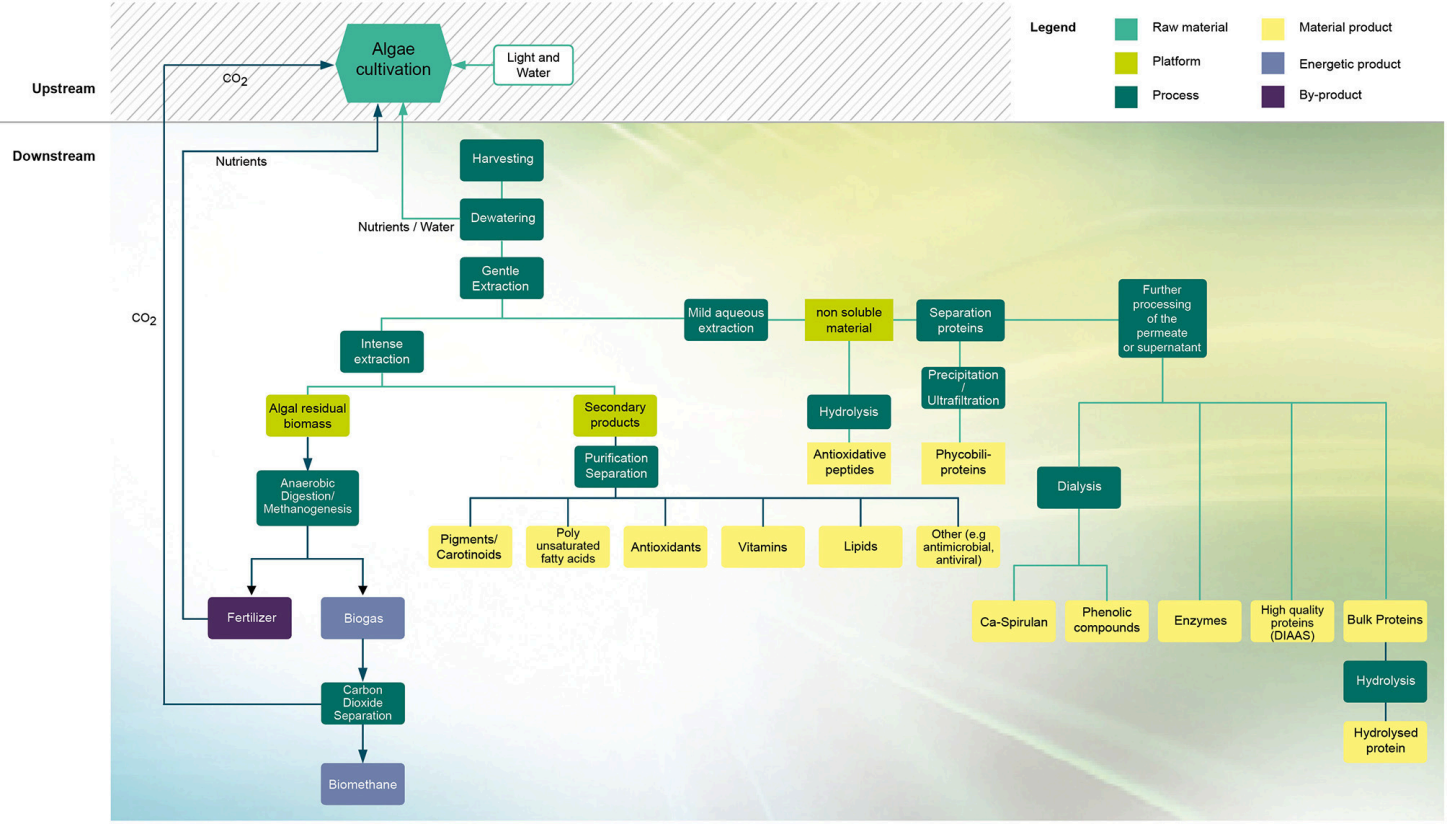
Biorefinery model for microalgae processing. Adapted from Mathys et al. (42). © 2013 Alexander Mathys All Rights Reserved.

Additionally, the economic conditions would also improve by developing sustainable biorefinery models to recover a range of products with food applications (Figure 1). Within the biorefinery approach, converting the residual biomass into biofuel could significantly contribute to reduce the biomass production costs. As an example, the anaerobic digestion process is an interesting alternative that generates biogas from microalgae residues (44). The biogas produced can be converted to biomethane and used as a biofuel for vehicles, and simultaneously, the aqueous stream generated, rich in nitrogen, can be used as fertiliser for microalgae cultivation (45, 46). The CO_2_ recovered from biogas upgrading could be used as a source of carbon for microalgae cultivation (46).

## Bioactive compounds as food supplements

Besides providing the organism with nutrients and energy for maintenance, growth, and physical activity, foods can be a vehicle for delivering bioactive compounds with health benefits. Proteins and peptides are one of the main groups of compounds showing biological and technological functionalities (47–49). Peptides contain up to 20–30 amino acids per molecule, remaining inactive within the primary structure of proteins until released either in the gastrointestinal tract when food is digested or during food processing, e.g., ripening, fermentation, cooking, or storage (47). These specific protein fragments can positively impact body functions or conditions, and may ultimately influence health. Peptides were first mentioned in 1950 as the cause of an enhanced bone calcification in rachitic infants (50) and steadily investigated afterwards, with milk proteins being one of the main sources (51). Antioxidative, antihypertensive, immunomodulatory, anticancerogenic, hepato-protective, and anticoagulant activities have been attributed to some macro and microalgae-peptides (52–56). Literature about peptides is scarce and the evidence on potential health benefits is not strong, with no evidence in humans being reported. However, the possibility of using macro- and microalgae peptides for innovative functional food products has become of great interest in recent years (17, 28–31, 41, 48, 56–60).

Among the different types of microalgae-derived compounds, those with antioxidant properties are probably the most interesting ones for industrial applications. Proteins and lipids in foods are prone to oxidation during industrial processing or storage; essential nutrients are destroyed and potentially toxic compounds are generated. Low-molecular weight off-flavour compounds produced during oxidation affect consumer acceptability. Even worse, potentially toxic products can strongly affect consumers' health by triggering chronic diseases such as cancer, arteriosclerosis, diabetes, coronary heart diseases, and neurological disorders (56). Solutions commonly applied in food processing to prevent oxidation include (i) minimising pro-oxidant content, i.e., substances that generate reactive oxygen species or inhibit antioxidant systems, such as free fatty acids, metals, and oxidised compounds; (ii) protecting foods from light; (iii) evacuating air or adding oxygen scavengers; and (iv) adding antioxidants (61). Some of the most common chemical antioxidants used in food industries since the 1970s include butylated hydroxyanisole (BHA) and butylated hydroxytoluene (BHT), EDTA, and others (57). Even when their utilisation is regulated by law and controlled, adverse effects on health have been reported for some synthetic additives (62). Consumers associate “synthetic” with unhealthy (63), leading their preferences towards more “natural” products (62).

Food industries have therefore had to evolve and adapt their technologies and products to meet consumer needs and demands, by reducing the utilisation of synthetic additives for example. With an increasingly popular “clean label” movement within the EU, interested in more “natural” and healthy foods free from additives, the possibility of labelling products guaranteeing the absence of synthetic additives is a key strategy for attracting consumers (63). Thus, natural additives claiming to be natural antioxidant preservatives are an attractive research field. Peptides with antioxidant or preservative properties can prolong food shelf life either by delaying or inhibiting oxidation (56, 57). So far, however, no results have been published on the utilisation of such microalgae-derived compounds in foods.

## Microalgae incorporation in foods with potential health benefits

It is possible to provide bioactive compounds to the majority of the population if added to foods that are widely accepted or regularly consumed. While the addition of peptides to foods has not been reported so far, other microalgae-derived compounds and the whole cells have been used as food ingredients with different purposes (Table 1). Raymundo et al. (64) and Gouveia et al. (65) observed positive effects on the techno-functional and the antioxidising properties of food emulsions when certain microalgae species were incorporated. Gels were suggested as a vehicle to provide valuable microalgae-based compounds (66–73). Batista et al. (66) incorporated several microalgae species into gels to improve their structure and as a way to provide antioxidants and certain ω-3 PUFAs to potential consumers. Similar studies but incorporating other microalgae species were reported by Gouveira et al. (67).

**Table 1:** Microalgae incorporation in different food products.

**Product**	**Microalgae incorporation**	**Addition**	**Benefit**	**References**
Oil/water emulsions	*C. vulgaris* green and *C. vulgaris* orange (after carotenogenesis)	2% w/w	Techno-functional properties	(64)
Oil/water emulsions	*C. vulgaris* green, *C. vulgaris* orange (after carotenogenesis) and *H. pluvialis* (red, after carotenogenesis)	*C. vulgaris*: 0.25–2.00% w/w *H. pluvialis*: 0.05–2.00% w/w	Colouring and nutritional properties (antioxidative activity)	(65)
Vegetarian food gels	*C. vulgaris, H. pluvialis, A. maxima* and *D. vlkianum*	0.75% w/w	Techno-functional and nutritional properties (antioxidative activity, ω-3 PUFAs).	(66)
Vegetarian food gels	*A. maxima* and *D. vlkianum*	0.1–% w/w	Techno-functional and nutritional properties (ω-3 PUFAs).	(67)
Vegetarian food gels	*H. pluvialis* and *A. maxima*	0.75% w/w	Techno-functional properties	(68)
Frozen yogurt	*Arthrospira* sp.	2–8% w/w	Nutritional properties	(70)
Dairy products (fermented milk)	*A. platensis*	3 g/L	Nutritional properties	(71)
Natural and probiotic yogurt	*A. platensis*	0.1–0.8% w/w	Techno-functional properties and nutritional properties.	(72)
Yogurt	*Chlorella* sp.	0.25% w/w extract powder and 2.5–10.0% extract liquid	Techno-functional properties and nutritional properties.	(74)
Processed cheese	*Chlorella* sp.	0.5 and 1.0% w/w	Techno-functional properties and nutritional properties.	(75)
Cookies	*C. vulgaris*	0.5, 1.0, 2.0, and 3.0% w/w	Colouring agent	(76)
Biscuits	*I. galbana*	1 and 3% w/w	Techno-functional properties and nutritional properties (ω-3 PUFAs)	(77)
Biscuits	*A. platensis*	*A. platensis*: 0.3, 0.6 and 0.9% Phycocyanin extract: 0.3% w/w to wheat flour	Nutritional properties	(78)
Biscuits	*A. platensis*	1.63, 3, 5, 7, 8.36% w/w	Techno-functional and nutritional properties (protein, fiber content and antioxidative activity)	(79)
Biscuits	*A. platensis, C. vulgaris, T. suecica* and *P. tricornutum*	2 and 6% w/w	Techno-functional properties and nutritional properties (antioxidative activity)	(80)
Cookies	*H. pluvialis*	Astaxanthin powder 5, 10, and 15% w/w	Techno-functional properties and nutritional properties (antioxidative activity)	(81)
Bread	*Dunaliella* sp.	Whole biomass, biomass after b-carotene extraction and, biomass after b-carotene and glycerol extraction: 10% w/w	Nutritional properties (protein content)	(82)
Bread	*O. amphibian and A. platensis*	5% w/w microalgae protein in flour	Techno-functional properties and nutritional properties	(83)
Bread	*A. fusiformis*	1 and 3% w/w in flour	Nutritional properties (proteins and mineral content)	(84)
Bread	*A. platensis*	11% w/w in flour	Techno-functional properties and nutritional properties (proteins and mineral content)	(85)
Bread	*Arthrospira* sp.	2, 2.5, and 3% w/w in flour	Nutritional properties (protein content)	(86)
Bread	*I. galbana, T. suecica, S. almeriensis*, and *N. gaditana*	0.47 % w/w in flour	Techno-functional properties	(87)
Gluten free bread	*A. platensis*	2, 3, 4, and 5% w/w in flour	Nutritional properties (proteins content)	(88)
Extruded snacks	*Arthrospira* sp.	0.4, 1.0, 1.8, 2.6, and 3.2% w/w	Techno-functional properties and nutritional properties (proteins content)	(89)
Pasta	*C. vulgaris* green, *C. vulgaris* orange (after carotenogenesis) and *A. maxima*	0.5, 1.0, and 2.0% w/w in flour	Techno-functional properties and nutritional properties	(90)
Pasta	*I. galbana* and *D. vlkianum*	0.5, 1.0, and 2.0% w/w in dry weight	Techno-functional properties and nutritional properties (ω-3 PUFAs)	(91)
Pasta	*A. platensis*	5, 10, and 20% w/w in flour	Techno-functional properties and nutritional properties (antioxidative activity)	(92)
Pasta	*D. salina*	1, 2, and 3% w/w in flour	Techno-functional properties and nutritional properties	(93)

Dairy products can also be incorporated with microalgae to deliver bioactive compounds (70). Several authors agree that certain species such as *Arthrospira* spp. can stimulate growth of desired probiotic bacteria in yogurts and fermented milk, increasing the viability of the probiotics (71). The availability of trace elements, vitamins, and other bioactive compounds in microalgae powders promotes the development of desired bacteria (71, 72). Previous studies suggested a synergy between microalgae and bacteria, where the former liberate exopolysaccharides into the medium that stimulate bacterial growth (73). *Chlorella* has been successfully incorporated into yogurts (74) and cheeses (75).

Cookies and biscuits are relevant categories to deliver microalgae-based ingredients. Reasons include good acceptance of taste, versatility, convenient consumption due to their ease of conservation and transportation, texture, and appearance. While *Chlorella vulgaris* has been incorporated into cookies as a colouring agent and also as a potential antioxidant and nutritional supplement (76), *Isochrysis galbana* was added to provide ω-3 PUFAs with beneficial effects for human health (77). Phycocyanin extracts and whole *A. platensis* were incorporated to produce cookies with potential health benefits (78) and to enhance protein and fibre content (79). Batista et al. (80) enhanced both nutritional and health benefits potential of cookies, i.e., increasing protein and antioxidants content, by incorporating *A. platensis, C. vulgaris, Tetraselmis suecica*, and *Phaeodactylum tricornutum*. Adding *Haematococcus pluvialis* in cookies increased the antioxidant capacities and lowered the glycaemic response (81). Like cookies, bread is also widely consumed. Some authors reported the incorporation of microalgae to enhance the nutritional properties of bread several years ago (82, 83). *Dunaliella* was suggested as a protein supplement years ago, being incorporated in white wheat bread (82). *Arthrospira* (83–85) and a decolourised extract obtained from this species (86) were also incorporated in bread to increase its protein content (83–86). Also other microalgae species were used in bread (87). Recent reports also mentioned the incorporation of microalgae in gluten-free bread (88); adding *Arthrospira* significantly increased protein content and improved bread quality due to the presence of some essential amino acids compared to non-supplemented bread. Similar benefits were observed when *Arthrospira* was used as ingredient in extruded snacks (89). Pasta is another widely accepted product. *C. vulgaris* and *Arthrospira maxima* both enhanced the nutritional content of fresh spaghetti, with the products being well accepted by a sensory panel (90). Pasta was also a vehicle to deliver ω-3 PUFAs (incorporation of *I. galbana* and *Diacronema vlkianum*) (91) and antioxidants with potential health benefits (92). Although *Arthrospira* increased the protein content, protein digestibility decreased as the microalgae content increased (92). An attempt to increase the nutritional value of pasta was made by adding *Dunaliella salina* powder to it; however, due to the low proportion of microalgae among the ingredients (below 3%) only a significant increase in minerals was observed (93). Only the incorporation of small quantities of microalgae and certain microalgae-derived products in foods has been reported so far, thereby these additions did not significantly enhance the macromolecular composition in foods, e.g., protein content. Some characteristics of microalgae limit their utilisation in food products. For example, despite the antioxidant-rich nature of *Chlorella* and *Arthrospira*, changes in colour and flavour in foods are usually perceived as undesirable by consumers (70, 75, 94). The green colour of microalgae limits its application in daily-use products, as it adversely affects consumers' perception about taste and quality (24). In products such as pasta, which is currently available on the market in different colours, consumers' perception is not affected by changes in colour; however, a slight fish flavour was negatively perceived in certain products upon adding microalgae (91).

The techno-functional properties determine the applicability of additives in food products. Properties such as emulsifying, foaming, gelation, water, and fat absorption capacities are reported for some microalgae proteins and hydrolysates, but many of them remain largely unknown (25, 58, 90, 95). Gouveia et al. (65) demonstrated that incorporating microalgae into emulsions allowed for a reduction in the percentage of oil, preserving its structure based on the possibility of microalgae to act as a fat mimetic, but only some vegetable proteins could be substituted without compromising the emulsion's stability. Emulsions' resistance to oxidation was enhanced. When incorporated into vegetarian desserts (protein-polysaccharides mixed gels) as colouring agents, the cell structure in microalgae protected pigments from thermal degradation during processing (67). Structural and rheological properties of gels were also improved, but these properties were species-dependent, mainly determined by lipids and microalgae proteins (66). In subsequent works, it was demonstrated that properties of gels are also linked to gel formulation and to the changes in the pH and composition derived from the salt content in microalgae (68, 69). *Chlorella* biomass decreased meltability and cohesiveness of processed cheese, but increased hardness and springiness (75). Incorporation into baked products such as cookies and bread resulted in a positive increased firmness (76–79). Microalgae are complex ingredients containing proteins, carbohydrates, and lipids amongst other compounds; addition into dough affects the internal structure of the dough due to the changes in water absorption or the incorporation of lipids (80). Cooking and textural properties of pasta were not affected by the addition of microalgae (90), but *Arthrospira* decreased the mechanical strength of raw pasta, which became more susceptible to breakage during handling (92). Additionally, a decreased gluten-protein content (when wheat flour was partially replaced) resulted in increased firmness, cohesiveness, and chewiness after cooking the pasta. A high concentration of microalgae increases the stickiness of pasta, whereas the elasticity is unaffected.

## Conclusions

Microalgae clearly show potential to meet the population's needs for more sustainable food solutions. The richness of compounds in microalgae can contribute to develop an algal-based food industry, focused on producing and utilising microalgae for innovative functional food products. Besides the protein content and balanced amino acids profiles, microalgae incorporation into foods could lead to potential benefits for human health due to the presence of bioactive compounds in some microalgae species. For example, antioxidative, antihypertensive, immunomodulatory, anticancerogenic, hepato-protective, and anticoagulant activities have been attributed to microalgae-derived peptides. Unfortunately, the utilisation of microalgae or microalgae-derived products as food substitutes is not competitive yet, mainly due to the low TRL and lack of economy of scale for microalgae cultivation and processing. Once these hurdles are overcome, incorporating microalgae as food ingredients will not only provide health benefits but will also contribute to improving issues related to sustainability, taking into account the growing population and our current diet, habits, and health.

## Author contributions

AM devised the main conceptual ideas. MC and AM discussed the ideas and commented on the manuscript. MC wrote the manuscript in consultation with AM.

### Conflict of interest statement

The authors declare that the present research was conducted with support of the Coop Research Program of the ETH Zurich World Food System Center (Grant number NewAlgae 2-72235-17), the Bühler AG, and ETH Zurich Foundation, Switzerland.

## Footnotes

^1^European Environment Agency (2017). Available online at: https://www.eea.europa.eu/data-and-maps/indicators/13.2-development-in-consumption-of-2/assessment-1 (Accessed March 19, 2018).

^2^Stice C, Basu A. (2015). Available online at: http://quarterly.luxresearchinc.com/quarterly/?p=139 (Accessed March 19, 2018).

^3^https://www.fda.gov/food/ingredientspackaginglabeling/gras/ucm083022.htm (Accessed March 19, 2018).
